# Infant pain vs. pain with parental suppression: Immediate and enduring impact on brain, pain and affect

**DOI:** 10.1371/journal.pone.0290871

**Published:** 2023-11-16

**Authors:** Gordon A. Barr, Maya Opendak, Rosemarie E. Perry, Emma Sarro, Regina M. Sullivan

**Affiliations:** 1 Department of Anesthesiology and Critical Care Medicine, The Children’s Hospital of Philadelphia, Philadelphia, Pennsylvania, United States of America; 2 Perelman School of Medicine at the University of Pennsylvania, Department of Psychology, University of Pennsylvania, Philadelphia, Pennsylvania, United States of America; 3 Child Study Center, Center for Early Childhood Health & Development, Child & Adolescent Psychiatry, New York University School of Medicine, New York, New York, United States of America; 4 Emotional Brain Institute, Nathan Kline Institute, Orangeburg, New York, United States of America; Technion Israel Institute of Technology, ISRAEL

## Abstract

**Background:**

In the short term, parental presence while a human infant is in pain buffers the immediate pain responses, although emerging evidence suggests repeated social buffering of pain may have untoward long-term effects.

**Methods/finding:**

To explore the short- and long-term impacts of social buffering of pain, we first measured the infant rat pup’s [postnatal day (PN) 8, or 12] response to mild tail shock with the mother present compared to shock alone or no shock. Shock with the mother reduced pain-related behavioral activation and USVs of pups at both ages and reduced Fos expression in the periaqueductal gray, hypothalamic paraventricular nucleus, and the amygdala at PN12 only. At PN12, shock with the mother compared to shock alone differentially regulated expression of several hundred genes related to G-protein-coupled receptors (GPCRs) and neural development, whereas PN8 pups showed a less robust and less coherent expression pattern. In a second set of experiments, pups were exposed to daily repeated Shock-mother pairings (or controls) at PN5-9 or PN10-14 (during and after pain sensitive period, respectively) and long-term outcome assessed in adults. Shock+mother pairing at PN5-9 reduced adult carrageenan-induced thermal hyperalgesia and reduced Fos expression, but PN10-14 pairings had minimal impact. The effect of infant treatment on adult affective behavior showed a complex treatment by age dependent effect. Adult social behavior was decreased following Shock+mother pairings at both PN5-9 and PN10-14, whereas shock alone had no effect. Adult fear responses to a predator odor were decreased only by PN10-14 treatment and the infant Shock alone and Shock+mother did not differ.

**Conclusions/significance:**

Overall, integrating these results into our understanding of long-term programming by repeated infant pain experiences, the data suggest that pain experienced within a social context impacts infant neurobehavioral responses and initiates an altered developmental trajectory of pain and affect processing that diverges from experiencing pain alone.

## Introduction

Human infants who are born premature or who are in intensive care are routinely exposed to many painful but medically necessary procedures [1, 2]. Infants are thought to feel this pain since the same brain circuitry required to perceive pain in adults is activated by noxious stimuli in newborn infants, with the exception of the amygdala and subareas of the prefrontal cortex (PFC) [3–6]. Thus, pain management is critical in newborns during painful yet often routine medical procedures. Due to complexities of managing infant pain pharmacologically and potential for untoward effects, non-pharmacological modes of pain reduction are being increasingly used.

The importance of pain management in the neonatal intensive care unit (NICU) goes beyond immediate pain relief for the infant since these repetitive painful medical procedures have enduring effects through “programming” of brain development [7–9] The pain-induced programming decreases the volume of the amygdala, a brain region that is particularly sensitive to perturbations in early life. Repeated prenatal exposure to increased stress hormones, or postnatal adversities, such as harsh parental care or pain, all involve amygdala-dependent activity and the sustained dysregulation of emotion as evidenced by outcome studies of children in the NICU [10, 11] and by models in rodents and nonhuman primates [11–17]. This programming impacts later-life pain processing and affect with long-lasting adverse outcomes for cognitive and emotional processing in both humans [18–22] and animal models [23–25]. Yet we do not know the consequences of repeated social buffering where the parent’s presence is paired multiple times with adverse events for the infant.

Across multiple species, a significant social partner can be a potent attenuator of pain and stress. This is termed social buffering [26, 27]. In altricial species, social buffering is particularly potent within the parent-infant dyad, occurring equally well with adoptive and biological parents indicating the importance of learning cues for social buffering during attachment formation [28]. The effectiveness of social buffering in soothing a stressed infant is illustrated by its use during routine medical visits for vaccinations, where parental presence decreases both the behavioral and stress hormone (cortisol) response to pain [29–31]. Social buffering is also used in most (~82%) USA NICUs for pain relief, including kangaroo care (skin-to-skin caregiver contact), tucking (caregiver holding baby’s head and bottom) and nursing/feeding/maternal odor [32–36]. These environmental interventions reduce the need for pharmaceuticals for pain relief and have the added benefit of comforting the caregiver [[33, 37–39], reviewed in [40]].

Despite the clinical use of social buffering, our understanding of mechanisms of pain reduction by important social cues remains incomplete. In adults, social buffering of pain and stress is associated with reduced systemic stress hormones and decreased activity of some core pain processing areas, including reduced activation pain circuitry including the anterior and posterior cingulate cortex, the insular and thalamus and increased activation of reward and safety-related brain regions such as the nucleus accumbens, caudate head, orbital frontal cortex, and ventral medial prefrontal cortex (mPFC) [[30, 41, 42], reviewed in [43]]. Even less is understood during early development, although considerable evidence suggests that the development of social buffering during the first couple of months of life sooths the child but without changes in stress hormones and without amygdala participation [5, 30, 44], which corresponds to postnatal rat pups younger than postnatal day (PN)10. In older infants, parental presence sooths the infant but also attenuates stress hormone release and blunts amygdala response in children [28], which is replicated in rat pups between the ages of PN10-15 [45, 46]. In animal models in the infant, it is the ventral tegmental area (VTA) that serves as the primary buffer of the amygdala during social buffering, rather than the PFC in adult rats and adult humans, highlighting a changing social buffering network [47–56]. Thus, the first goal of these experiments is to examine the ability of the mother to buffer the infants response to noxious shock and to define changes in the activation of neural circuits and amygdala gene expression during the pain sensitive period and the attachment sensitive period.

Finally, across species, this social buffering process is disrupted in infants who have experienced repeated trauma with parents, as evidenced by less calming and an attenuated decrease of stress hormones and amygdala suppression [30, 57, 58]. This has been replicated in infant rodents, where mechanisms can be explored. For example, repeated experience with trauma within a social context (i.e. maltreatment) degrades social buffering’s calming effect, as well as blocking stress hormone and amygdala activity reduction, which is replicated by repeated pain in an anesthetized mother’s presence but not when pup is alone [48, 56, 59, 60]. Furthermore, only pain within a social contact disrupts amygdala structure and function, but not by pain when the pup is alone [56, 60]. Thus, repeated pain experienced by the infant in a social context initiates a unique neural network compared to pain alone. What is not known are the long-term effects of repeated experiences of social buffering in the infant. Non-social, non-pharmacological reduction in infant pain with repeated exposure to sucrose, although effective in reducing short-term pain in humans and rodent models [61–63] may have deleterious long term consequences [64–66]. Therefore, a second goal of this work is to examine changes in adult responsiveness to pain and social behavior following repeated maternal buffering of infant pain, including activation of neural circuits following inflammatory pain in the adult.

The approach of the present series of experiments on the social vs. nonsocial consequences of infant pain that is buffered or not by the maternal presence was two-fold. First, we focused on the immediate effects of social buffering of pain on an expanded neural network during infancy, going beyond the amygdala. Second, we assessed the long-term outcome of repeated pain within a social context on later life pain and socio-emotional domains in adults. We used two age ranges: 1) PN5-9, during a sensitive period of pain’s long-term programming [49, 67–73]; and 2) PN10-14, during the limited age range when social buffering of pups’ stress response by the mother diminishes pups’ response to pain and blocks the amygdala’s participation in the response to pain [45, 46, 74–78]. We hypothesized that repeatedly shocking infants would alter inflammatory pain responses in adults most robustly when experienced PN5-9 (pain sensitive period), whereas pain experiences during PN10-14 (robust social buffering) would impact affective behaviors more broadly. Following infant treatment with and without the mother, these hypotheses were tested in adults in response to pain (basal thermal responses and inflammatory thermal hyperalgesia) and two affective behaviors, the unlearned response to threat and the response to a social figure.

### General design

**Fig 1:**
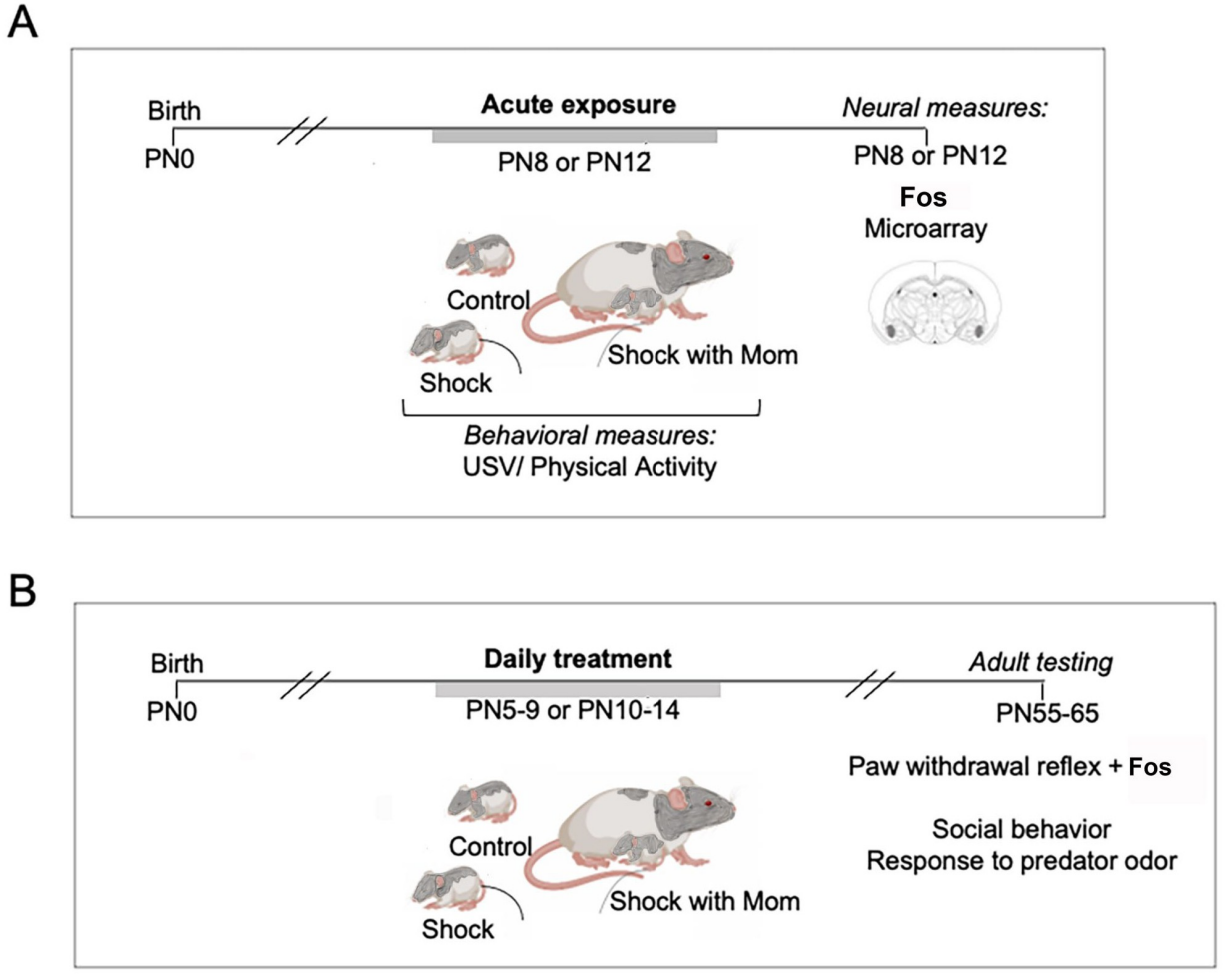
Schematic of experimental design. **A.** *Acute treatment*. At PN8 or PN12, pups received nine mild tail shocks (1-sec 0.5mA tail shock, each shock separated by a 4-min interval) without the mother (Shock alone), with the mother (Shock+mother), or were placed in the treatment container and not shocked (Control). During treatment, we assessed their behavioral activity and USV’s and Fos expression in the PAG, amygdala, and PVN of the hypothalamus. In a separate cohort of animals, we assess changed changes in gene expression induced in the amygdala by the three treatment conditions at both ages. **B,** *Chronic treatment over 5 days*. Pups were shocked with or without the dam or were not shocked each day for 5 days during the pain sensitive period (PN5-9) or during an age range when the mother’s presence suppresses activation of the amygdala during shock (PN10-14). Adult rats were tested for basal pain thresholds using the thermal plantar test and on the next day for inflammation induced hyperalgesia, with brains removed for Fos after this test. In separate cohorts of adult animals, we assessed affective behaviors using a test for social behavior towards a conspecific and threat-induced behaviors with presentations of a predator odor. *Key*: USVs- ultrasonic vocalizations; PAG- periaqueductal gray; PVN- periventricular nucleus.

Fig 1A illustrated the acute one-day procedure at either PN8 (± 1 day) or PN12 (± 1 day). Fig 1B illustrates the design for the Chronic 5-day treatment from PN5-9, the sensitive period of pain which is prior to the functional maturation of the amygdala, or PN10-14, the transitional sensitive period when the maternal presence begins to blunt the HPA, reduce the response to pain, and attenuate amygdala response to shock.

## Methods and materials

### Subjects

We used male and female Long-Evans rats (ENVIGO) born and bred in the NKI colony. Animals were housed in polypropylene cages (34 x 29 x 17 cm) with an abundance of wood shavings for nest building and kept in a 20°C environment with a 12:12 light-dark cycle. Food and water were available *ad libitum*. The day of birth was considered PN0, and pups from 133 litters were culled to a maximum of 12 pups (6 males and 6 females as possible) on postnatal day 1. Those reared to adulthood were housed in two/cage in same sex and treatment cages and handled regularly until tested. The Institutional Animal Care and Use Committees at both institutions approved all animal care and experimental procedures (protocols, CHOP: 16–001008; NYU: TR202200004), and follow the guidelines from the National Institutes of Health.

*Subject ages for infant treatment*. 1), Acute (one-day procedure—see Fig 1A) at either PN8 (+/- 1day) or PN12 (+/- 1day); or 2) Chronic (5 days) treatments from PN5-9, the sensitive period for pain and prior to functional maturation of the amygdala or PN10-14, the transitional sensitive period when maternal presence begins to blunt the HPA, reduce the response to pain and attenuate amygdala response to shock (Fig 1B).

### Procedures for infant shock treatment

*Infant shock with and without maternal presence*. Infant rat pups were assigned to one of three treatment groups typically with one male and one female from each litter represented in each of the treatment conditions: shock alone (“Shock”), shock with maternal presence (“Shock+mother”) or no shock/no mother (“Control”). Importantly, to limit trauma to our experimental manipulations, care was taken not to disrupt the mother and induce trampling and/or rough retrieval of pups by the mother as pups were taken or returned to the nest. If this occurred, pups were not used. However, there were no systematic changes in the mother’s behavior due to the infant pain experience [see [56]]. Pups that received shock were outfitted with a wire electrode on the tail for automated shock delivery and placed within Plexiglas training apparatus (taking approximately 5min for 8 pups). Pups with maternal presence had a Urethane anesthetized mother present; Urethane prevents the milk ejection reflex and thus pups did not receive milk. All pups were kept at a thermoneutral body temperature through ambient and surface heat (surgical heater) heat within 0.1°C. Following a 10-minute acclimation period to recover from experimental handling, pups designated to receive shock had nine presentations of a 1-sec 0.5mA tail shock, each shock separated by 4-minutes. During the acute infant sessions ultrasonic vocalizations (USV) and behavioral activation (automatic measurement of pixel changes, Ethovision) were assessed in the infants [79–81]. The shock produced no physical damage to the pups and there was zero mortality, and although pup behaviors were recorded, at this age these behaviors are limited to increases/decreases in activity, approach/move-away from the mother, borrowing and probing the mother. It is therefore difficult to measure more sophisticated processes such as emotions. Pups with both an awake and an anesthetized mother show social buffering of the stress response, and it is unclear if pups can distinguish between an anesthetized mother and a sleeping mother. Following testing, pups were euthanized by rapid decapitation and the brains removed and frozen (~2min). For Fos studies, pups were kept in a quiet, dark thermoneutral areas for 60 min, then deeply anesthetized followed by brain removal.

#### Microarray

Immediately following the acute tests of shock with and without the dam, pups were euthanized, as were Controls at the same time. The infant brains were rapidly frozen (-45oC—total time from decapitation to frozen ~ 2min), amygdala dissected, stored in a (-80oC) freezer and processed for total RNA (Qiagen RNeasy Micro Kit). mRNA was amplified linearly and hybridized to Affymetrix rat 2.0 gene arrays as specified by Affymetrix (Santa Clara, CA) and NuGEN (Pico System; San Carlos, CA).

#### Fos IHC

Fos staining was conducted as previously described [82–84] at infant ages PN8 or PN12 or as adults following the carrageenan-induced thermal hyperalgesia. Following treatment, adult animals were housed in a quiet, dark room and pups kept in a warmed incubator for 30-min (~90-min after the start of testing). Pups were deeply anesthetized and euthanized followed by brain removal. Adults were deeply anesthetized and perfused with saline followed by 4% paraformaldehyde before brain removal. For all ages, brains were sectioned on a cryostat and sections placed on a microscope slide for cresyl violet or Fos staining, using a modified protocol of the avidin-biotin-peroxidase system [67, 85–87]. The sections were incubated for 24–48 hours in the primary antibody, rabbit anti-Fos (Santa Cruz) diluted 1:2,000 in PBS with Triton-X and 1% goat serum. Sections were stained using diaminobenzidine peroxidase substrate tablets (Sigma, Saint Louis, MO).

Fos analysis for pups focused on three regions known to be responsive to both pain and the mother during infancy—the periaqueductal gray of the midbrain (PAG), amygdala and paraventricular nucleus of the hypothalamus (PVN) [67, 85, 86]. Tissue from controls and the treated animals were processed together.

### Procedures for adult testing

At PN55-65, adult rats with infant treatment (e.g., PN5-9 or PN10-14 exposure to shock with and without the mother and controls) were tested for basal levels of thermal pain and on the next day for carrageenan induced thermal hyperalgesia, or separately for affective behaviors (fear, social behavior). Separate cohorts of animals were used for the pain, fear, and social behavior tests.

#### Adult pain baseline testing

On day one, a plantar thermal test was used [88] for baseline latencies and carrageenan induced hyperalgesia. Withdrawal responses were tested on each hindpaw three times and withdrawal latencies averaged over paw and trials.

#### Carrageenan induced hyperalgesia

On the day after basal testing, carrageenan was injected to the plantar pad of one hindpaw (1%, 50 μl) and one hour later withdrawal responses were again tested on each hindpaw three times and withdrawal latencies averaged over trials. The latencies of the injected and the contralateral hindpaws were averaged separately.

#### Social behavior tests

An adaptation of the Crawley three chamber test apparatus was used to test social behavior [89]. Animals were acclimated to the test apparatus for 5-minute on the previous day. On the day of testing, a slightly smaller same sex animal was placed into a perforated metal boxes (6x6 cm) in one chamber and an empty box in the other chamber. The test animal was then given a 10-minute test and time spent in each chamber, the duration of nose contact with target animal, and activity level were recorded using the Noldus Ethovision system, to determine the animal’s location.

#### Fear behavior to predator odor tests

The day before testing, all animals were given a 5-min familiarization to a large box divided into three equal sized chambers connected by small portals to permit the animal free access. This permitted animals to learn the location of a hiding compartment (a small hutch) in the first chamber. On test day, the 3 chambers apparatus was composed of the same small hiding hutch, the center was left empty (in which the animal was placed at the start of the test), and the third chamber contained predator odor (fox urine, Pete Rickard’s Red Fox Urine Hunting Scent (1 mL) placed on a Kimwipe in one of the corners. Rats were able to approach/interact with the Kimwipe by direct sniffing, biting, or moving the predator odor about the testing arena. They were also able to move away from the area that contained the Kimwipe or “hide” in the chamber distant from the odor. Data were analyzed using Ethovision to determine the rat’s location.

#### Fos IHC analysis

Similar procedures were used on pups and adults, although adults were perfused before brain removal. All brains were harvested 90 minutes after the carrageenan-induced hyperalgesia and focused on the pain network, including PFC (infralimbic; prelimbic), cingulate cortex, nucleus accumbens (core and shell), amygdala (BLA and medial), hypothalamic (anterior, paraventricular nucleus, preoptic, and ventral medial area), thalamus (paraventricular nucleus and central nucleus combined), and the periaqueductal gray (ventrolateral, lateral, and dorsal regions). Structures were identified from the Nissl-stained sections and compared to those in several different brain atlases. Sections were examined at 4X magnification and the regions of interested outlined. Photomicrographs were taken of the region of interest and counted using ImageJ. All counting was done blind to the experimental conditions.

### Statistical analysis

#### Behavior and Fos

All behavioral outcomes and Fos counts were analyzed by one-way ANOVA’s (infant treatment) followed by posthoc tests using Tukey’s statistic to correct for multiple comparisons, except for the thermal withdrawal data from both paws (S1 Fig) which was a two-way ANOVA. We were not powered to assess sex differences for the behavioral data, but preliminary analyses showed no consistent sex differences and data from female and male subjects were combined. N’s were sufficiently small for the Fos and microarray data that sex comparisons were not done.

#### Microarray data analysis

Microarray analyses were like those previously published by our group [68, 90, 91]. Male and female data were combined and not analyzed separately. Expression values were preprocessed, background-corrected, and normalized by RMA [92, 93] using BRAINARRAY [University of Michigan; CDF: Rat 1.0_Rn_ENTREZG; [94]] resulting in approximately 19,000 distinct genes. Rank Products [95], which simulates probabilities based on pairwise comparison differences, was used to assess differential expression in each experimental condition compared to the other. Here we focus on functional analyses between the shock with and without mother conditions.

To determine significant gene ontology (GO) categories for the entire data set we input in all genes with the fold change or pfp values (percentage of false positive predictions) calculated by the Rank Products analysis into ErmineJ using the gene score resampling method (GSR), which assesses the contribution of all ~19,000 genes to the ontologies [https://erminej.msl.ubc.ca; [96]]. Default settings were used except that we used 200,000 iterations and only examined Biological Process GO categories. In some cases, we input top genes into DAVID [(http://david.abcc.ncifcrf.gov/) [92, 97]] for further classification.

To define networks of related probes from the comparison between shock with and without the mother, we used Ingenuity Pathways Analysis (IPA; Ingenuity Systems, www.ingenuity.com) inputting probes with fold change that exceeded ±1.25, using the direct connection option. We included up- and downregulated genes together because the analytic methods in IPA can generate pathways from both and because of the biological relevance of up- and down-regulated genes. The IPA Functional Analysis identifies the biological functions most significant to the dataset. For each dataset analyzed in this manner, the functional pathways analysis identified predefined pathways from the Ingenuity Pathways Analysis library that were most significant to the dataset. The association between the dataset and the canonical pathway was measured by Benjamini-Hochberg’s test for the probability that the association between the genes in the dataset and the canonical pathway was not by chance. We assessed the top three networks at each age using canonical paths from IPA database to identify coherent networks.

## Results and discussion

### Infant data

**Fig 2:**
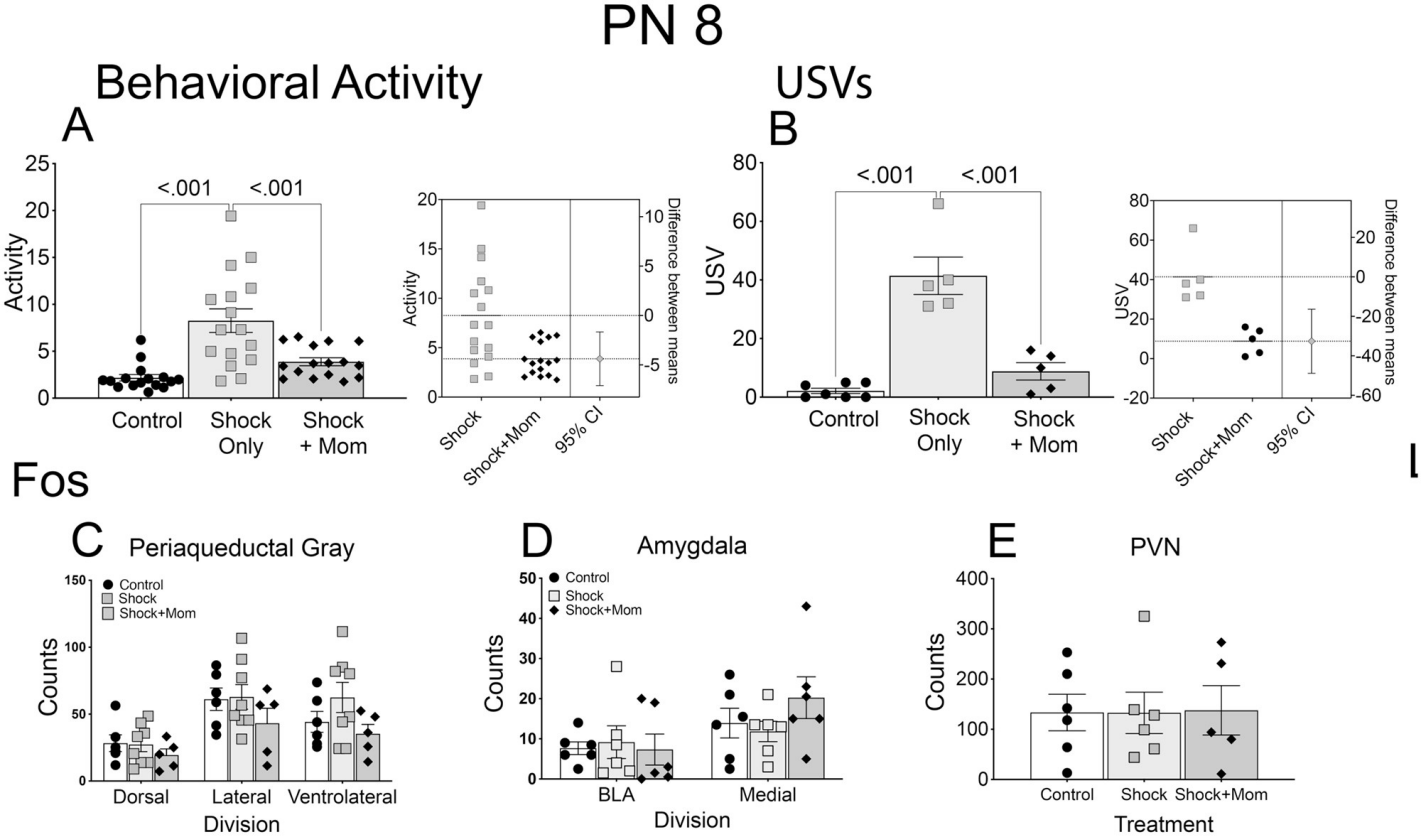
Infant treatment at PN8: Shock induced behavioral activation and Fos expression with and without the dam compared to controls. Shock induced behavioral activity (A; measured as in [90] and USV’s (B) were decreased by the mother’s presence during shock exposure. Estimation plots, to the right of the data figures confirm the robustness of these effects. Despite the behavioral changes there were no concomitant alterations in Fos expression in the PAG (C), amygdala (D), or PVN (E). N = 15–16 for Activity; N = 5–7 for USV; and N = 5–6 for Fos. *Key*: USVs- ultrasonic vocalizations; PAG- periaqueductal gray; BLA- basolateral amygdala; PVN- periventricular nucleus of the hypothalamus.

**Fig 3:**
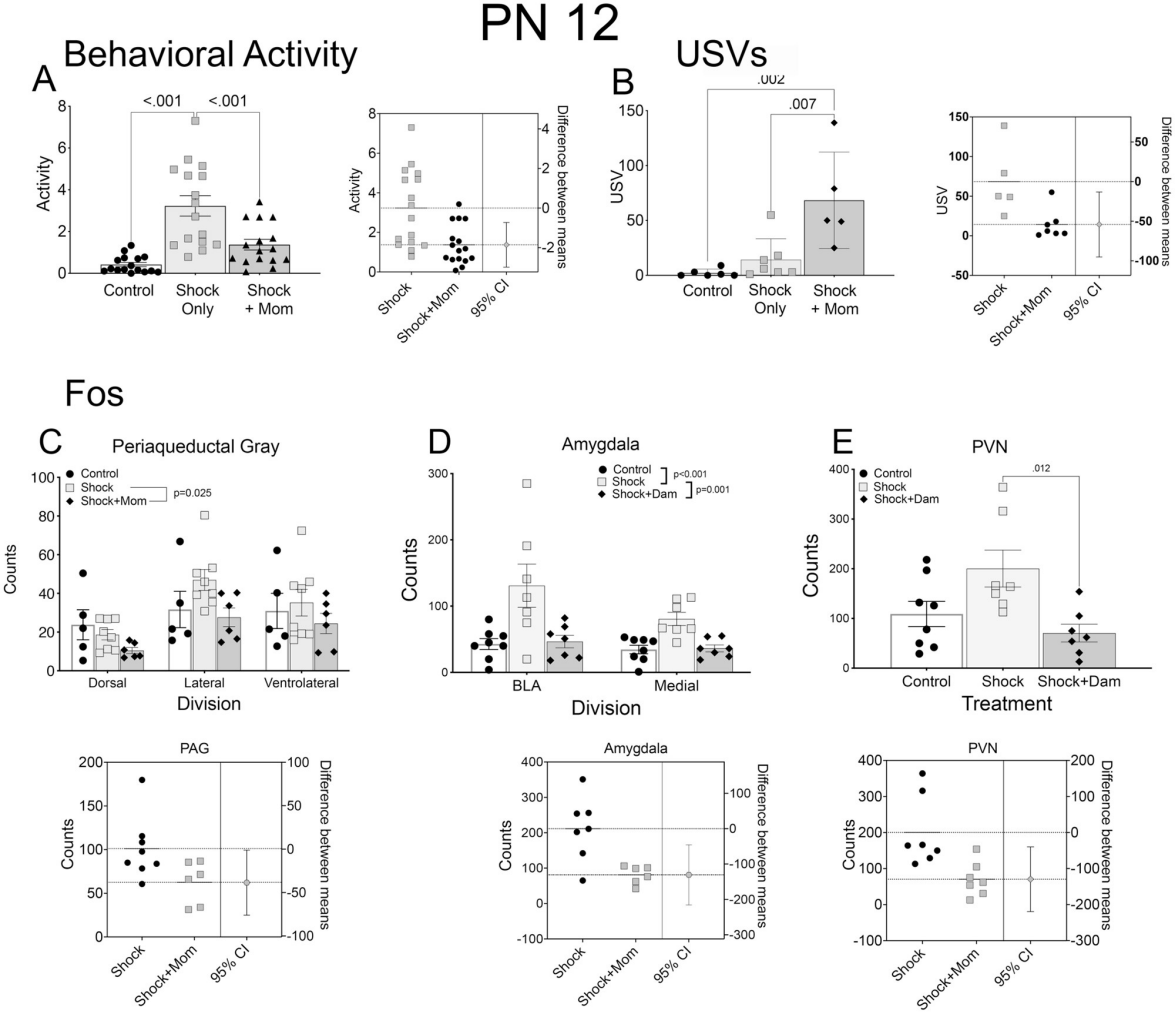
Infant treatment at PN12: Shock induced behavioral and Fos expression changes with and without the dam compared to controls. Shock induced behavioral activity (A; measured as in (81)) and USV’s (B) were decreased by the mother’s presence during shock exposures, similarly to the younger PN8 pups. Estimation plots, to the right of the data figures confirm the robustness of these effects. Fos expression (C) differed between the Shock alone and Shock+mother for all three brain areas and in addition between the Control and the Shock alone conditions. Estimation plots for each brain area are shown below the data plots. N = 15–17 for Activity; N = 5 for USV; and N = 5–6 for Fos. *Key*: USVs- ultrasonic vocalizations; PAG- periaqueductal gray; BLA- basolateral amygdala; PVN- periventricular nucleus.

*Behavioral response to shock with and without the mother*. At both PN8 (Fig 2A and 2B) and PN12 (Fig 3A and 3B), the presence of the mother significantly decreased behavioral activity to shock [PN8, F(2,45 = 15.71, p<0.001; PN12, F(2,15) = 19.50; p<0.001] and decreased USV’s to shock [PN8, F(2,14) = 32.97, p<0.001; PN12, F(2,15) = 10.10, p < 0.002].

#### Fos immunohistochemistry

At PN8, there was no changes in Fos expression in any of the three measured regions in the Shock alone condition and no effect of the mother (PAG: p’s>0.164 for the three regions; amygdala: p’s>0.322 for the two subnuclei; PVN; p = 0.996; Fig 2C–2E). In contrast, at PN12, there was an increased shock induced Fos expression in the three subdivisions of the PAG, the BLA and medial amygdala and the PVN, and each was reduced by the presence of the dam (all F values > 5.00; p’s<0.020; Fig 3C–3E).

#### Microarray

For presentation purposes we focused on comparisons between shock with and without the mother’s presence, although data from all three groups can be found in Table 1 that provides an overview of the levels of significance for all comparisons. For all data, upregulation is the Shock alone>Shock+mother conditions; downregulation is Shock alone<Shock+mother.

**Table 1:** Number of significant genes and fold change.

Absolute Fold	Shock vs Shock+mother	Control vs Shock+mother	Control vs shock
**PN8**			
*2*.*50*	1	0	1
*2*.*00*	2	0	1
*1*.*50*	12	3	4
*1*.*25*	137	57	79
**Pfp**			
.*001*	42	26	19
.*01*	79	46	62
.*05*	161	128	146
.*10*	228	209	203
**PN12**			
*2*.*50*	2	3	3
*2*.*00*	19	18	25
*1*.*50*	114	99	151
*1*.*25*	690	570	643
**Pfp**			
.*001*	20	16	50
.*01*	64	54	196
.*05*	185	126	389
.*10*	298	206	546

*Note*. Pfp (percent of false positives) is the probability from the Ranked Products analysis. The counts include both up- and down-regulated genes.

#### Microarray—Differential expression

Our initial efforts were to determine differentially expressed probes using the Ranked Products analysis. Table 1 shows the number of differentially regulated genes at PN8 and PN12. At PN8, there were few fold changes greater than 1.50 and minimum effects of the mother on the number of changed probes. At PN12, there were about three times as many altered probes, measured by either fold-change or pfp values and substantially more affected probes in the Shock alone condition compared to controls. In addition, the presence of the mother reduced the number of probes significantly at all pfp levels and to a lesser extent for the fold change at PN12 but not at PN8. Thus, in agreement with prior results [68, 90], there is a stronger effect of the shock on the amygdala gene expression at PN12 than at PN8, and a greater effect of the mother on that gene expression at that age. These list of significant probes (p<. 01) are in Tables 2 and 3.

**Table 2:** Significant probes at PN8: Shock vs. Shock+mother.

Entrez Gene	Symbol	Gene Name	pfp	Fold
299219	Gle1-ps1	GLE1 RNA export mediator homolog (yeast), pseudogene 1	0.0000	1.62
287314	Olr1416	olfactory receptor 1416	0.0000	1.52
290655	Crlf1	cytokine receptor-like factor 1	0.0000	1.44
499586	Tmem212	transmembrane protein 212	0.0000	1.43
1003140390	Mir154	microRNA 154	0.0000	-1.32
171138	Kcne2	potassium channel, voltage-gated Isk-related subfamily E regulatory beta subunit 2	0.0000	-1.33
100314283	Mir377	microRNA 377	0.0000	-1.34
170496	Lcn2	lipocalin 2	0.0000	-1.34
100314168	Mir379	microRNA 379	0.0000	-1.35
100314128	Mir544	microRNA 544	0.0000	-1.36
100360437	Nsun3	NOP2/Sun domain family, member 3	0.0000	-1.36
100314167	Mir539	microRNA 539	0.0000	-1.37
171049	Folr1	folate receptor 1 (adult)	0.0000	-1.38
100314040	Mir181b1	microRNA 181b-1	0.0000	-1.39
100314085	Mir382	microRNA 382	0.0000	-1.43
100314105	Mir384	microRNA 384	0.0000	-1.46
94341	Kcnj13	potassium channel, inwardly rectifying subfamily J, member 13	0.0000	-1.48
65129	Cldn1	claudin 1	0.0000	-1.53
294696	Mrps36	mitochondrial ribosomal protein S36	0.0000	-1.61
300920	Cldn2	claudin 2	0.0000	-1.67
363069	Ppcdc	phosphopantothenoylcysteine decarboxylase	0.0000	-1.68
266803	Sostdc1	sclerostin domain containing 1	0.0000	-1.70
315597	Mfrp	membrane frizzled-related protein	0.0000	-1.94
83504	Kl	Klotho	0.0000	-2.00
304081	Clic6	chloride intracellular channel 6	0.0000	-2.66
24856	Ttr	Transthyretin	0.0000	-5.15
298702	Icam4	intercellular adhesion molecule 4, Landsteiner-Wiener blood group	0.0010	1.39
291328	Slc39a12	solute carrier family 39 (zinc transporter), member 12	0.0010	1.37
64640	Rpl30	ribosomal protein L30	0.0010	-1.24
252897	Pcdhga11	protocadherin gamma subfamily A, 11	0.0010	-1.28
100313970	Mir325	microRNA 325	0.0010	-1.29
362424	Tmem72	transmembrane protein 72	0.0010	-1.36
500926	Or10ad1	olfactory receptor, family 10, subfamily AD, member 1	0.0020	1.44
100270683	Tas1r2	taste receptor, type 1, member 2	0.0020	1.41
360572	Rpl23a	ribosomal protein L23a	0.0020	1.31
497794	Mug1	murinoglobulin 1	0.0020	-1.26
304277	Cpsf4	cleavage and polyadenylation specific factor 4	0.0020	-1.28
396527	Ugt1a2	UDP glucuronosyltransferase 1 family, polypeptide A2	0.0020	-1.29
100314079	Mir543	microRNA 543	0.0020	-1.30
84050	Enpp2	ectonucleotide pyrophosphatase/phosphodiesterase 2	0.0020	-1.31
25240	Aqp1	aquaporin 1	0.0020	-1.33
80900	Slco1a5	solute carrier organic anion transporter family, member 1a5	0.0030	-1.26
100314173	Mir380	microRNA 380	0.0030	-1.29
292548	Zfp418	zinc finger protein 418	0.0040	-1.35
289230	Nhlh1	nescient helix loop helix 1	0.0050	1.37
100359826	Krtap8-1	keratin associated protein 8–1	0.0050	1.35
117249	Dnah3	dynein, axonemal, heavy chain 3	0.0060	1.33
684028	Cryga	crystallin, gamma A	0.0060	-1.33
100362431	Ttc30a	tetratricopeptide repeat domain 30A	0.0060	-1.35
100313969	Mir323	microRNA 323	0.0070	-1.27
293347	Gvinp1	GTPase, very large interferon inducible 1	0.0080	1.36
100314111	Mir495	microRNA 495	0.0080	-1.25
303163	Igtp	interferon gamma induced GTPase	0.0080	-1.34
100314284	Mir487b	microRNA 487b	0.0090	-1.20
246172	Nexn	nexilin (F actin binding protein)	0.0090	-1.28
685055	Atr	ATR serine/threonine kinase	0.0090	-1.29
24825	Tf	Transferrin	0.0100	-1.16
297386	Slc4a5	solute carrier family 4, sodium bicarbonate cotransporter, member 5	0.0100	-1.19
362141	Slc38a11	solute carrier family 38, member 11	0.0100	-1.20
684623	Gpr52	G protein-coupled receptor 52	0.0100	-1.21
294687	Bdp1	B double prime 1, subunit of RNA polymerase III transcription initiation factor IIIB	0.0100	-1.26

**Note:** The lists in Tables 2 (above) and 3 (below) do not contain hypothetical gene descriptions and thus the number of probes listed here differs slightly from that of Table 1. Fold is Shock compared to Shock+mother. Upregulation is Shock>Shock+mother; downregulation is Shock<Shock+mother.

**Table 3:** Significant probes at PN12: Shock vs. Shock+mother.

Entrez Gene	Symbol	Gene Name	pfp	Fold
29708	Gabra6	gamma-aminobutyric acid (GABA) A receptor, alpha 6	0.000	2.511
29458	Neurod1	neuronal differentiation 1	0.000	2.455
365748	Bhlhe22	basic helix-loop-helix family, member e22	0.000	2.245
81737	Ntf3	neurotrophin 3	0.000	2.193
305719	Fezf2	Fez family zinc finger 2	0.000	2.113
500137	Neurod6	neuronal differentiation 6	0.000	1.898
171387	Rnf39	ring finger protein 39	0.000	1.813
29527	Ptgs2	prostaglandin-endoperoxide synthase 2	0.000	1.566
296188	Sptlc3	serine palmitoyltransferase, long chain base subunit 3	0.000	-1.853
305309	Slc10a4	solute carrier family 10, member 4	0.000	-1.887
25085	Th	tyrosine hydroxylase	0.000	-2.048
29310	Mc3r	melanocortin 3 receptor	0.000	-2.054
498335	Cxcl13	chemokine (C-X-C motif) ligand 13	0.000	-2.097
25569	Trh	thyrotropin releasing hormone	0.000	-2.098
296866	Gpr165	G protein-coupled receptor 165	0.000	-2.184
317608	Gpr101	G protein-coupled receptor 101	0.000	-2.237
309888	Sim1	single-minded family bHLH transcription factor 1	0.000	-2.298
64444	Isl1	ISL LIM homeobox 1	0.000	-2.372
65191	Gabre	gamma-aminobutyric acid (GABA) A receptor, epsilon	0.000	-2.424
116506	Calcr	calcitonin receptor	0.000	-2.504
499947	Cbln4	cerebellin 4 precursor	0.001	-1.962
500048	Fezf1	Fez family zinc finger 1	0.001	-1.886
302819	Arhgap36	Rho GTPase activating protein 36	0.001	-2.262
54276	Neurod2	neuronal differentiation 2	0.001	2.043
29227	Nfib	nuclear factor I/B	0.001	1.763
294789	Ranbp3l	RAN binding protein 3-like	0.001	1.453
78974	Six3	SIX homeobox 3	0.001	-1.741
85426	Slc5a7	solute carrier family 5 (sodium/choline cotransporter), member 7	0.001	-1.706
296867	Pgr15l	G protein-coupled receptor 15-like	0.001	-1.816
367745	Dgkk	diacylglycerol kinase kappa	0.001	-1.982
65187	Gabrq	gamma-aminobutyric acid receptor, theta	0.001	-1.993
24318	Drd2	dopamine receptor D2	0.001	-1.783
500017	Asb4	ankyrin repeat and SOCS box-containing 4	0.001	-1.810
315350	Irs4	insulin receptor substrate 4	0.001	-1.952
313453	Tmem255a	transmembrane protein 255A	0.002	-1.781
60417	Ecel1	endothelin converting enzyme-like 1	0.002	-1.917
499566	Car13	carbonic anhydrase 13	0.002	-1.493
60422	Slc18a3	solute carrier family 18 (vesicular acetylcholine transporter), member 3	0.002	-1.539
100362205	Fndc9	fibronectin type III domain containing 9	0.002	-1.784
64619	Zfp238	zinc finger protein 238	0.002	1.776
24553	Met	MET proto-oncogene, receptor tyrosine kinase	0.002	1.592
361412	Adamts18	ADAM metallopeptidase with thrombospondin type 1 motif, 18	0.003	1.769
117097	Gpr50	G protein-coupled receptor 50	0.003	-1.639
25526	Ptgds	prostaglandin D2 synthase (brain)	0.003	1.420
299757	Nts	Neurotensin	0.003	-1.794
291819	Car7	carbonic anhydrase 7	0.004	1.922
305509	Adcy1	adenylate cyclase 1 (brain)	0.004	1.645
58853	Nr4a3	nuclear receptor subfamily 4, group A, member 3	0.004	1.603
100302373	Ctxn2	cortexin 2	0.004	-1.840
192251	Gpr149	G protein-coupled receptor 149	0.004	-1.624
361875	Atp6ap1l	ATPase, H+ transporting, lysosomal accessory protein 1-like	0.005	-1.662
114587	Dlk1	delta-like 1 homolog (Drosophila)	0.005	-1.860
300458	Npsr1	neuropeptide S receptor 1	0.005	-1.922
100313995	Mir9-2	microRNA 9–2	0.006	1.736
307829	Zfhx3	zinc finger homeobox 3	0.006	-1.681
116676	Aldh1a2	aldehyde dehydrogenase 1 family, member A2	0.006	1.418
25269	Pvalb	Parvalbumin	0.007	1.590
64317	Gpx3	glutathione peroxidase 3	0.008	-1.803
192649	Prokr2	prokineticin receptor 2	0.009	1.376
295041	Stoml3	stomatin (Epb7.2)-like 3	0.009	-1.261
25051	Glp1r	glucagon-like peptide 1 receptor	0.009	-1.680
294795	Capsl	calcyphosine-like	0.010	-1.559
25665	Scn5a	sodium channel, voltage-gated, type V, alpha subunit	0.010	-2.026

**Note**: The lists in Tables 2 and 3 do not contain hypothetical gene description and thus the number of probes listed here differs slightly from that of Table 1. Fold is Shock compared to Shock+mother. Upregulation is Shock>Shock+mother; downregulation is Shock<Shock+mother.

#### Microarray—Functional analysis

To assess further the functional classification of the differentially expressed probes, we used ErmineJ to test for over-enrichment of gene ontology categories [96]. For both ages, we input the fold or the pfp data. The results using both types of input values were very similar and thus we only present the fold-change results. We also only discuss Biological Processes of the GO analysis.

There were no significant functional groups for the 8-day old pups (Table 4). Additional functional cluster analysis with DAVID confirmed this. For the PN12 pups, and the shock with and without the dam, there were 14 GO categories that were significant (corrected p<0.010). These spanned a range of biological functions, including nervous system development, but also cyclic nucleotide regulation and signaling. Table 4 shows the GO categories for PN12.

**Table 4:** Gene ontologies altered: Shock vs Shock/Mother.

PN8	*NONE DETECTED*		
***PN12***			
**GO ID**	***Ontology/Neurodevelopment***	**Number of Genes**	**Corrected P-Value**
GO:0021987	cerebral cortex development	73	4.34E-10
GO:0021978	telencephalon regionalization	9	4.78E-10
GO:0021871	forebrain regionalization	19	5.31E-10
GO:0021797	forebrain anterior/posterior pattern specification	5	5.97E-10
GO:0021796	cerebral cortex regionalization	6	6.82E-10
GO:0021766	hippocampus development	47	7.96E-10
GO:0021761	limbic system development	65	9.55E-10
GO:0021542	dentate gyrus development	10	1.19E-09
GO:0003407	neural retina development	39	4.21E-03
GO:0001764	neuron migration	83	4.48E-03
GO:0021954	central nervous system neuron development	52	6.82E-03
GO:0021954	central nervous system neuron development	52	6.82E-03
GO:0021536	diencephalon development	53	7.16E-03
GO:0021895	cerebral cortex neuron differentiation	14	7.27E-03
**GO ID**	***GPC Receptors and Signaling***	**Number of Genes**	**Corrected P-Value**
GO:0007218	neuropeptide signaling pathway	57	2.39E-09
GO:0007187	G-protein coupled receptor signaling pathway, coupled to cyclic nucleotide second messenger	72	4.78E-09
GO:0030817	regulation of cAMP biosynthetic process	52	3.41E-03
GO:0007188	adenylate cyclase-modulating G-protein coupled receptor signaling pathway	66	3.98E-03
GO:0030814	regulation of cAMP metabolic process	62	6.97E-03
GO:0001505	regulation of neurotransmitter levels	95	7.64E-03
GO:0060078	regulation of postsynaptic membrane potential	28	8.53E-03
GO:0030819	positive regulation of cAMP biosynthetic process	33	8.84E-03
GO:0006836	neurotransmitter transport	64	9.06E-03
**GO ID**	***Other***	**Number of Genes**	**Corrected P-Value**
GO:0031016	pancreas development	44	5.03E-03
GO:0048593	camera-type eye morphogenesis	80	6.51E-03
GO:0001709	cell fate determination	26	9.19E-03
GO:0014031	mesenchymal cell development	64	9.55E-03

#### Microarray—Ingenuity analysis

To determine pathways altered in the Shock alone vs. the Shock+mother conditions, we input all genes whose fold was greater that ±1.25 into the Ingenuity IPA core analysis program using the direct connection option. This provided networks identified as over-representations of functional networks and associated canonical paths for the Shock alone compared to the Shock+mother condition.

**Fig 4:**
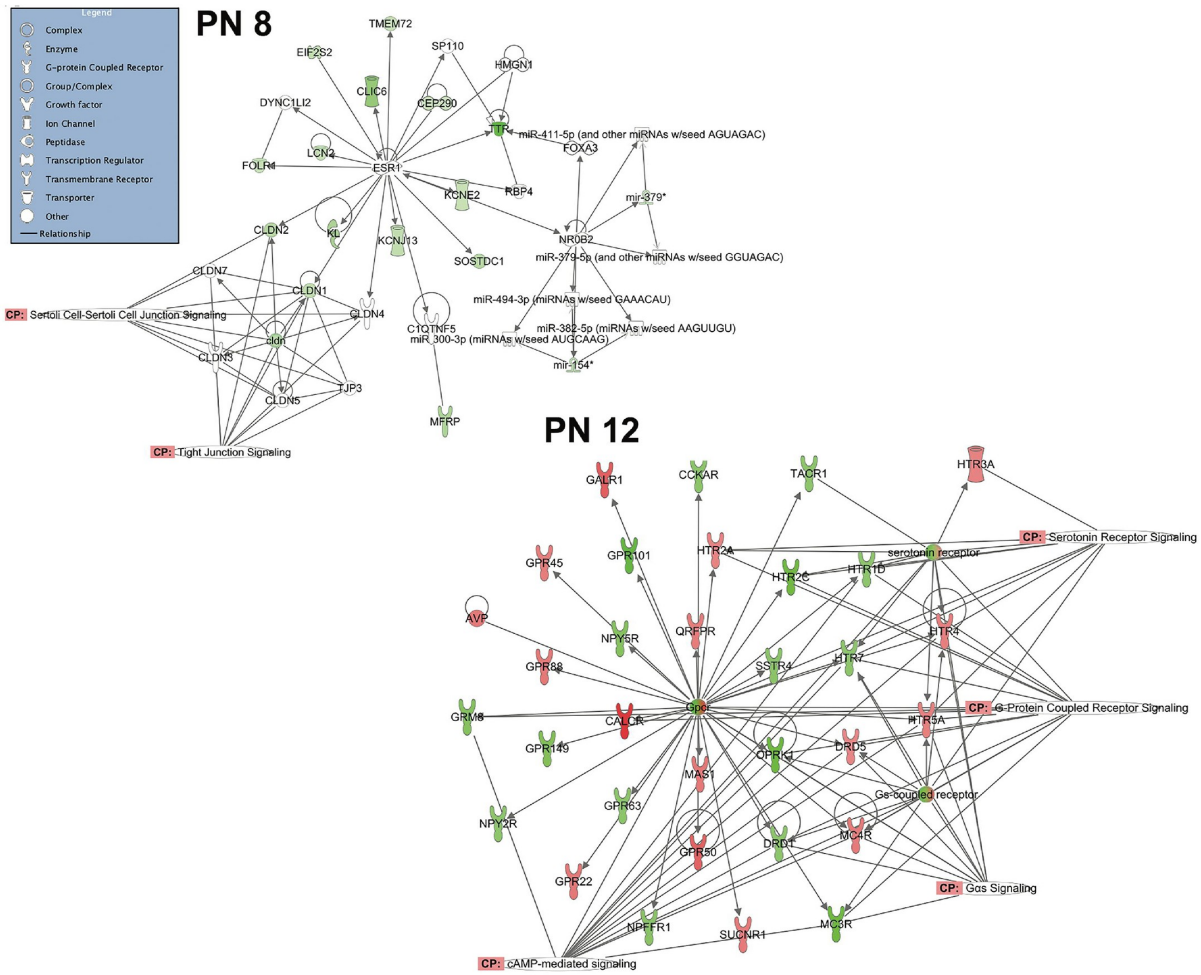
Infant network core analysis from IPA. The legend in the box at the top left corner defines the shapes used in the networks. In these networks, green is downregulated (Shock < Shock+mother) and red is upregulated (Shock > Shock+mother). The top left network shows the results from PN8. No canonical paths were significant at the normal level of stringency; those shown are with reduced stringency and account for a small cluster of genes. In contrast there were several significant paths at PN12 (lower right), focused on GCPRs and signaling. These results are consistent with those from the ErmineJ analysis that found no significant GO categories at PN8 but G-protein related function at PN12 (Table 4). For the older animals, changes were largely in receptors, whereas for the younger pups, there were a more diverse set of processes changed. N = 4 for each group and age. Key: IPA- Ingenuity Pathways Analysis; GPCRs- G-protein-coupled receptors; GO-Gene ontology.

At 8 days of age when the shock with and without mother conditions were analyzed, there were no significant over-represented canonical pathways (Benjamin-Hochberg correction, all p’s>.10), consistent with the results of the ErmineJ and DAVID results. With relaxed criteria, there were changes in inflammatory paths (not shown). Network analysis was consistent with this in that the main pathways in the first network were inflammatory paths that were downregulated by the presence of the mother (Fig 4).

At PN12, the four significant canonical paths included: 1) serotonin signaling; 2) G-protein-coupled receptors (GPCR)-mediated integration of enteroendocrine signaling; 3) transcriptional regulatory network in embryonic stem cells; and 4) G-protein coupled receptor signaling. Network analysis showed association of genes that formed paths focused around GPCR-coupled signaling, and cAMP-mediated signaling. One path included GPCR’s for dopamine, calcitonin, serotonin, galanin, Neuropeptide Y, metabotropic glutamate, and kappa opioids, among others (Fig 4), consistent with prior work showing activation of dopamine and its D1 receptor at this age by the mother [56, 68]. A second path showed similar GCRP-signaling paths and included kainate receptors, mu opioid receptors, opioid peptides including prodynorphin and proenkephalin, and other peptide gene receptors including CCK and TRH. In addition, genes related to neurodevelopment and differentiation were differentially regulated (e.g., NeuroD, Slit2, St18, Nkx22), consistent with the results from the ErmineJ analyses (Table 4).

#### Basal thermal thresholds

**Fig 5:**
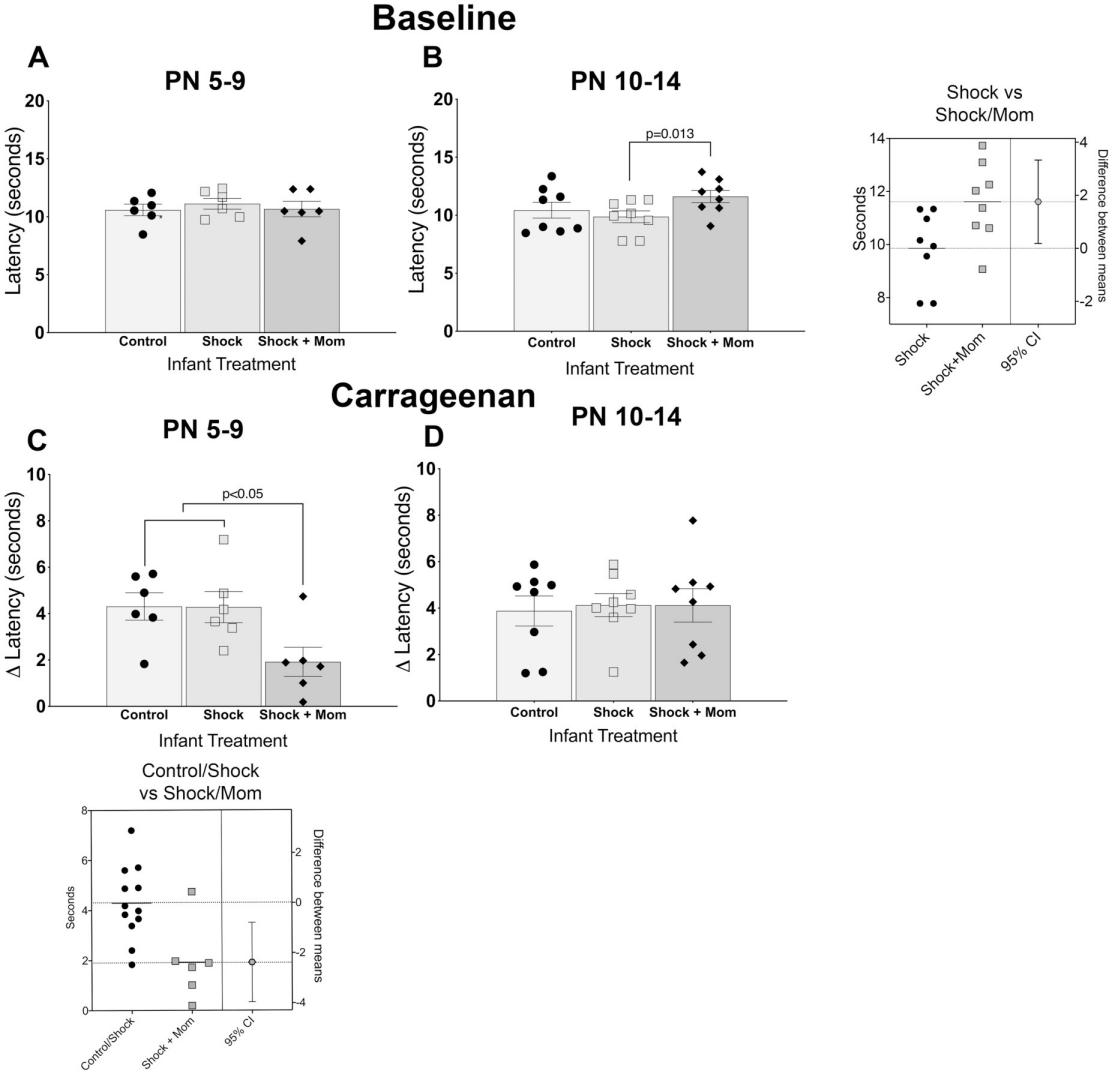
Adult thermal withdrawal latencies either at baseline. Adults were tested for baseline responses (A,B) or one hour following intraplantar injection of carrageenan (C, D). There was a small but significant hypoalgesia when adults had been treated in the infant Shock+mother condition at PN10-14 as compared to those receiving Shock alone. An estimation plot shown to the right of panel B, comparing the Shock+mother group to the control and shock groups suggests that despite the small difference, the effect was reliable. Graphs C and D show the degree of adult hyperalgesia (baseline-carrageenan latencies), which is the decrease in withdrawal latencies from baseline to the carrageenan response. Following infant PN5-9 treatment, adults decreased their withdrawal latencies for the Control and Shock groups by slightly more than 4 seconds, whereas for the Shock+mother infant treatment, it was less than two seconds (B). The estimation plot below the graph shows the reliable effect. There were no reductions in the hyperalgesia in the injection paw if the adult had been exposed to shock with or without the mother as infants at PN10-14 (D). N = 6–8 for each group and age.

Data from the three trials and both paws were averaged. There was a small but significant elevation in the thermal withdrawal latency in the Shock+mother pups that were treated at PN10-14 [F(2,14) = 4.16, p = 0.038; Fig 5C), but not PN5-9 [F(2,10 = 0.22, p = 0.807; Fig 5A). The estimation plot for the PN10-14 confirmed the effect.

#### Carrageenan induced hyperalgesia

For the younger treated animals, two escaped the test chamber during testing and roamed free until re-captured. Testing was not continued for them and the data from those animals are not included in the analyses. Carrageenan induced hyperalgesia for animals treated at both ages: the response latency was shorter for the inflamed paw than for the contralateral side. When the latencies of both the injected and contralateral sides in the treated animals were analyzed, there was a significant difference between the injected and the contralateral paws (PN5-9: F(1,15) = 92.30;p<0.001; PN10-14: F(1,21) = 124.00; p<0.001) and a significant treatment X paw interaction for the younger but not older treated animals (PN5-9: F(2,15) = 4.72, P = 0.026); PN10-14: F(2,21) = 0.050, P = 0.952); See S1 Fig

In contrast to the baseline results, there was a lessening of the thermal hyperalgesic effect induced by carrageenan in pups exposed to shock with the mother at PN5-9, but not PN10-14. When the difference scores were analyzed, there was significant lessening of the hyperalgesic effect in the injected paw compared to the Shock group and the untreated Controls (PN5-9: F(2,15) = 4.72, p = .026; PN10-14: F(2,21) = 0.050; p = 0.952 Fig 5B and 5D). The reduction was slightly more than 2 seconds compared to the baseline of about 10 seconds and represents a reduction by half of the hyperalgesic response from a more rapid 4 second withdrawal response to a two second withdrawal. Although the magnitude of the change is small, it is similar to that reported in other studies that show a similar difference when studying the analgesic properties of cannabinoids, for example [e.g. [98]]. Thus, the mother’s effect on basal and inflamed responses differed by age of treatment.

**Fig 6:**
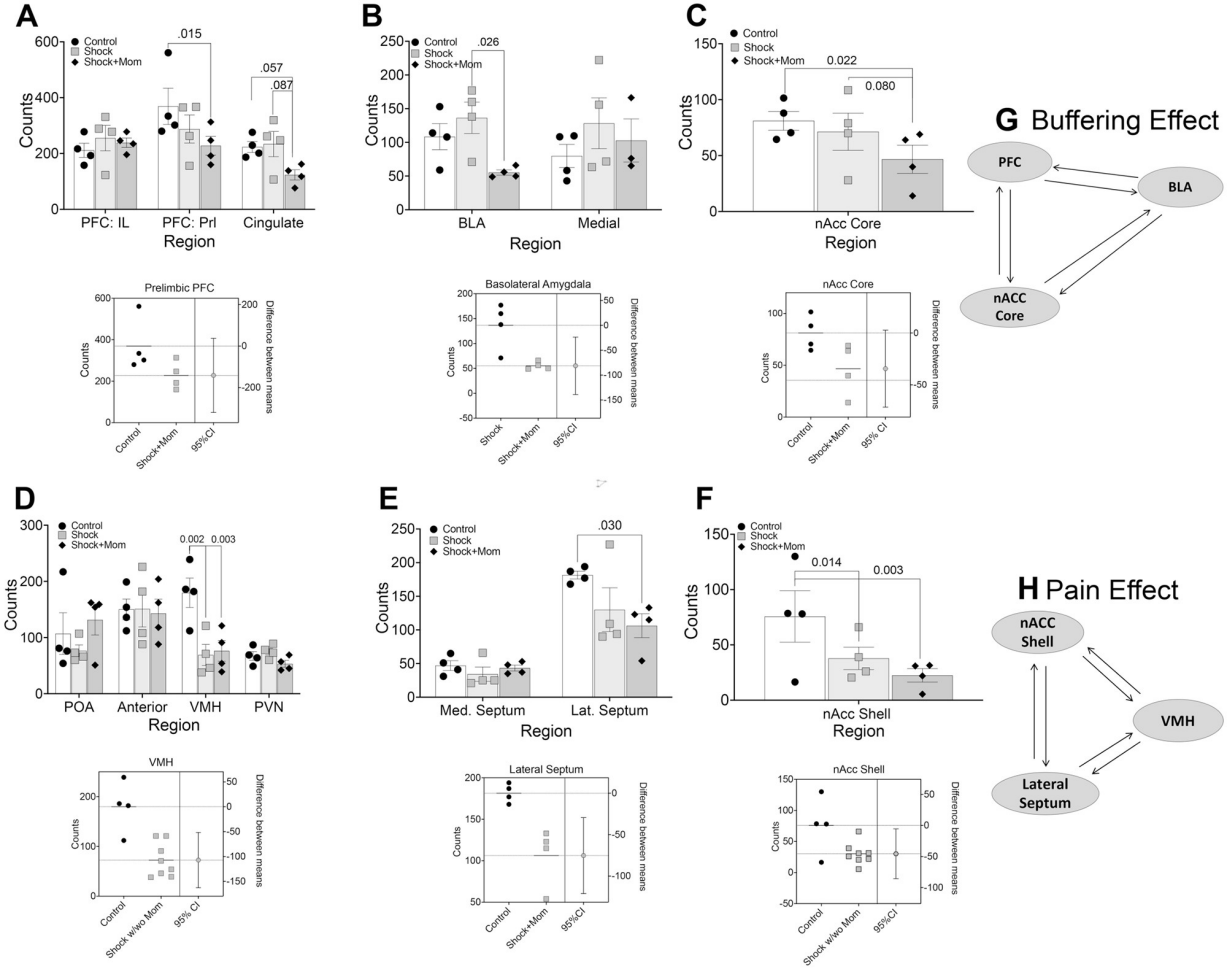
Adult Fos expression in the PN5-9 group tested as adults for carrageenan-induced hyperalgesia. There were two distinct patterns of change. The top row represents the pattern where the dam’s presence reduced Fos expression. We labeled this the “Buffering” effect (G). For these regions, there were significant reductions in Fos expression in Shock+mother condition in the prelimbic PFC (A), BLA (B), nAcc core (C), and VMH. Estimation plots are shown below the data panels. The second pattern, the “Pain” effect (H), was the reduction in Fos expression in both the Shock alone group and Shock+mother group and was seen in the VMH (D), the lateral septum €, and the nAcc shell (F). Again, estimation plots are below. For the VMH and nAcc shell we included data from both the Shock group and Shock+mother group (shock w/wo mother), since both were significant. N = 4 per region.

As shown in Fig 6, Fos expression after the carrageenan-thermal test was primarily assessed in adults that had been trained at PN5-PN9, since only those animals showed changes in carrageenan induced hyperalgesia. Posthoc analysis following the omnibus ANOVA’s for the younger exposed rats showed a significant reduction in Fos expression for the Shock+mother paired group compared to the Shock alone group in the cingulate cortex, nAcc core and basolateral amygdala. Fos expression was reduced by shock with or without the mother in the prelimbic PFC, nAcc shell, lateral septum and VMH. There were no differences in the thalamus or PAG (S2 Fig).

#### Behavioral assessment of affective behaviors following infant pain experiences with and without the mother

Affect is a critical feature of the response to pain and for this reason, we also performed some general assessment of adult affective behaviors as determined by response to response to a social figure or a threat. These data complement our recent assessment of infant pain and altered social behavior in infancy, altered by disrupted dopamine from the VTA to the basolateral amygdala [56].

#### Social behavior

**Fig 7:**
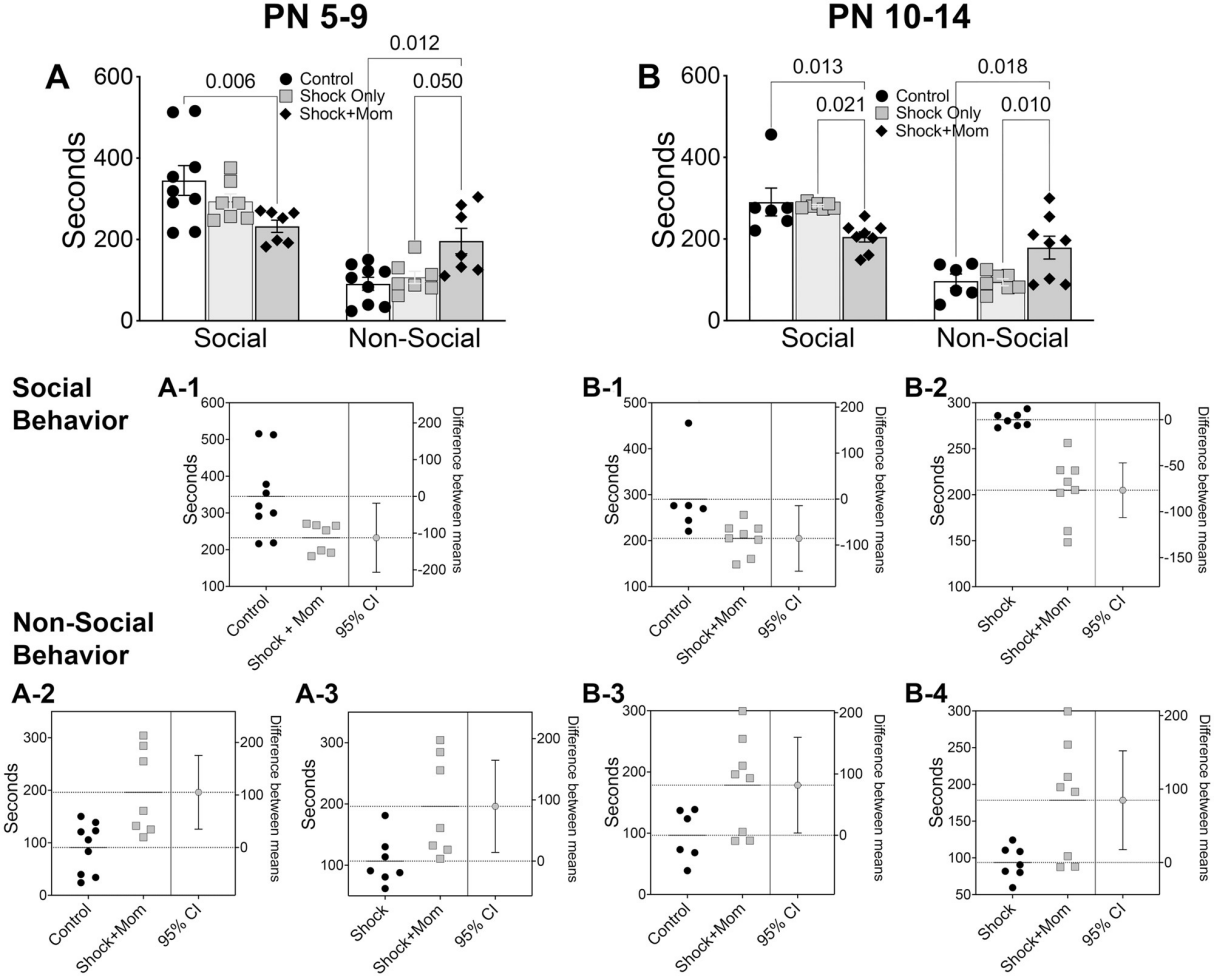
Adult social behavior to a conspecific. Social behavior (time spent investigating the conspecific) was reduced and non-social behavior (time spent in the chamber away from the conspecific) was increased by the infant Shock+mother treatment (**A, B**). This was independent of age at which Shock+mother exposure occurred. Shock alone had no effect. The estimation plots show the confidence intervals for social behavior (**A-1, B-1, B-2**) just below the data slides, whereas similar plots for the non-social behavior (**A-2, A-3, B-3, B-4**) are presented in the bottom four panels. N = 6–8 per condition.

As shown in Fig 7, at both training ages, pups shocked with the mother and tested as adults, showed less time with the stimulus animal (PN5-9: F(2,20) = 4.14, p = 0.031; PN10-14: F(2,18) = 6.61, p = 0.007) and more time in non-social chamber (PN5-9: F(2,10) = 7.01, p = 0.005; PN10-14: F(2,18) = 5.56, p = 0.013) than did the Control or Shock alone groups. Specifically, *posthoc* tests showed a significant difference between controls and Shock+mother group for social behavior and between Shock+mother and the Control and Shock alone groups for non-social behavior for both age groups. Thus, infant pain-mother treatments at both ages disrupted social behavior regardless of the age of exposure.

#### Unlearned fear

**Fig 8:**
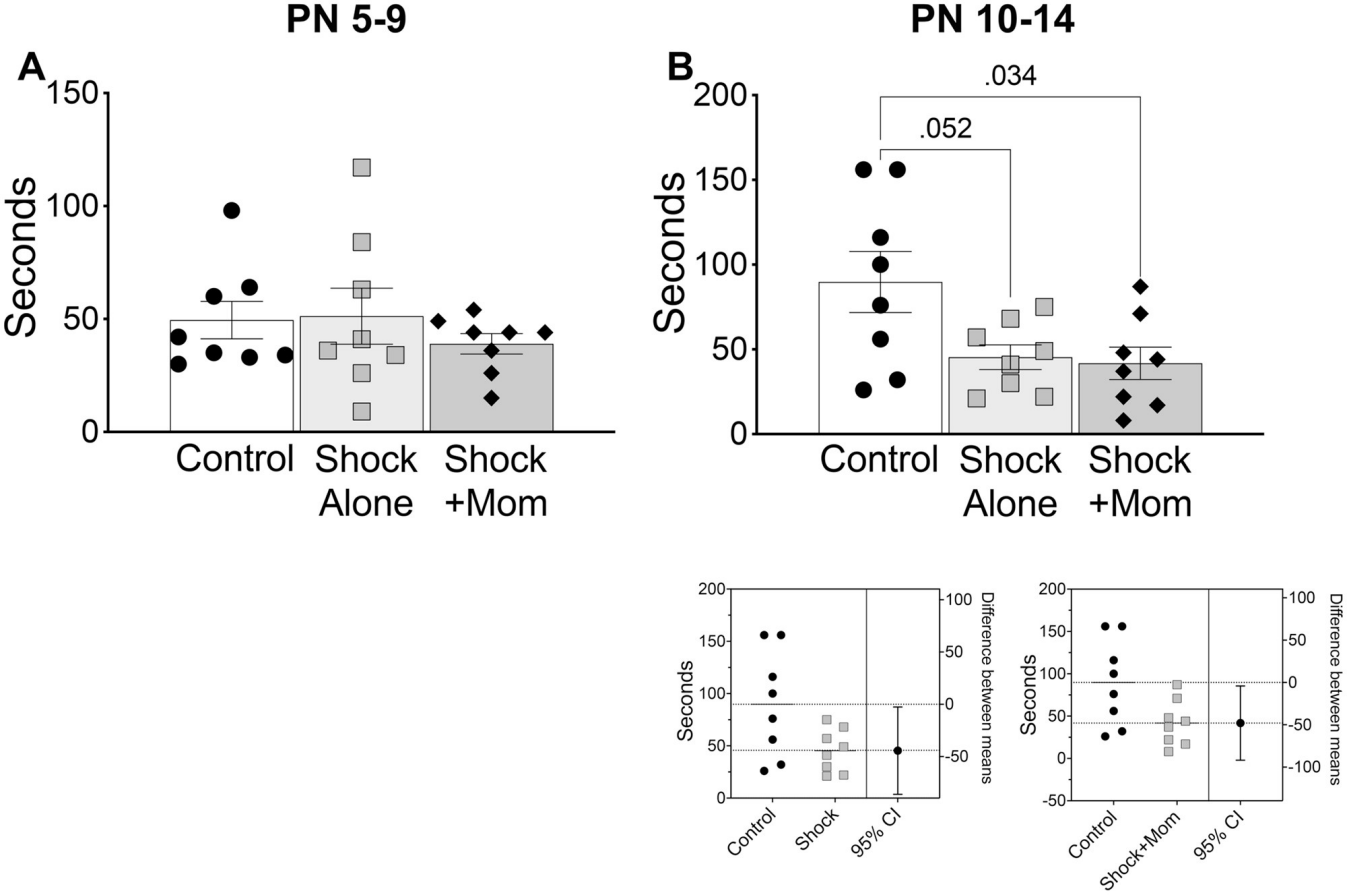
Adult hiding to a predator odor. **(A)** Adult testing following PN5-9 Shock+mother or Shock alone infant treatment had no effect on adult fear response to predator odor as measured via time spent in a hide box away from the threatening stimulus. (B) Adult testing following PN10-14 treatment reduced fear in both Shock+mother and Shock alone, as evidenced by reduced time spent hiding away from the predator odor, relative to controls. Estimation plots are below for the comparison of Control to Shock and of Control to Shock+mother. N = 8 for each group and age.

As shown in Fig 8, only PN10-14 infant treatment altered adult response to threat: both Shock alone and Shock+mother groups were significantly less likely to engage in hiding when exposed to a threat compared to controls [PN5-9 (F(2,21) = 0.54, p = 0.589; and PN10-14 (F(2,21) = 4.56, p = 0.023].

## Discussion

Our results, summarized in Table 5, illustrate the importance of social context during infant pain as guiding the infant’s responses to the mother and initiating an altered developmental trajectory and adult neurobehavioral outcome. At PN8 and PN12, the presence of the mother reduced the pain-related behavioral responses to the shock to similar extents, but only at PN12 did we observe activation of the amygdala, PAG and PVN by shock and its reduction by the mother’s presence. Moreover, parental modification of amygdala gene expression was organized around developmental and neurotransmission processes only in the older PN12 pups. Thus, in the young PN8 rat pup, the behavioral calming effects of the mother must be organized neurally by other paths, perhaps the locus ceruleus norepinephrine system, which is altered by maternal presence and well-documented to be important for attachment and learning about the mother [69], although this needs to be tested.

**Table 5:** Summary of results.

***Acute Treatment***	***Behavior***		***Fos Expression***			
Acute shock PN8	Decreased activity, USV with mother		No changes			
Acute Shock PN12	Decreased activity, USV with mother		Reduced Fos in PAG, BLA, PVN with mother			

***Chronic Treatment***	***Pain related behaviors***		***Affect related behaviors***		***Fos Expression after hyperalgesia***	
	*Baseline Thermal Latencies*	*Hyperalgesia*	*Social Behavior*	*Fear to predator*	*Fos changed with mother*	*Fos changed by shock*
PN9-5	No effect	Greatly reduced in mother treated	Decreased in mother treated	No effect	PFC (PRL, cingulate), BLA, NAcc (core)	VMH, NAcc (shell) lateral septum
PN10-14	Slight hypoalgesia in mother treated	No Effect	Decreased in mother treated	Decreased by both mother and shock	Not determined	

The long-term consequences for our adult outcomes following repeated infant pain also varied by age of infant treatment. As illustrated in Fig 5, we found reduced responsiveness to thermal pain in the adult after PN10-14 Shock+mother treatment (sensitive period for complete blockade of amygdala during social buffering), but not if the shock was experienced alone. In contrast, for the same Shock+mother treatment in the younger pups (PN5-9; sensitive period for pain programming), the adult outcome was decreased hyperalgesia when the noxious stimulus was applied to the inflamed paw. This younger rodent treatment age range is thought to correspond to that of premature infants in the NICU. Adult Fos assessment following nociceptive testing of brain areas known to be involved in pain and affective processing were specifically altered by the Shock+mother treatment, including the PFC (Prl, Cingulate), BLA, and nAcc core. Other areas changed regardless of the social context of pain, including the VMH, lateral septum and nAcc shell. These data are discussed below.

Since affective responses to pain are critical components of the pain experience in adults [99–102], we assessed adult social behavior and the response to a threat (predator odor). At both treatment ages, Shock+ mother treatment but not Shock alone resulted in decreased social behavior, whereas the response to threat was decreased only in PN10-14 treatment regardless of the social context (both Shock and Shock+mother treatment groups). These data point to the importance the context in which pain and injury are experienced, the age at which tissue injury occurs, and the nature of the adult affect assessed.

### Infant effects of acute pain and social buffering on brain-Fos

The neural activation as measured by Fos expression showed developmental divergence of circuitry activated by shock: at PN8, experiencing Shock+mother and Shock alone did not alter neural responsiveness in the PAG, BLA, or PVN but at PN12 Fos expression in the PAG, BLA, or PVN was decreased to near control levels when the mother was present compared to Shock alone. These data are a partial replication of the maturation of Fos in the brain following acute inflammatory injury: PAG and PVN activity following a formalin injection into the hindpaw, was absent at PN3 and did not appear until PN14, the next age tested [67]. These findings suggest that the behavioral activation by pain and the reduction in behavior by the mother are mediated by a different circuits in the younger pups, likely the locus ceruleus/olfactory bulb/piriform cortex circuitry [103].

In human newborns and adults receiving comparable noxious pinpricks, 18 of the 20 adult brain areas identified by fMRI associated with pain were also activated in the infant. Notably, two brain areas functional in the adult but not activated in the human newborn were the amygdala and orbitofrontal cortex of the PFC [5]. In young rat pups, <PN10, pain did not activate the amygdala either, but this did not prevent pups from responding behaviorally to injury, mediated by other structures at that age [56]. This lack of amygdala engagement in the neural network for threat and pain has also been reported in nonhuman primates and humans [46, 49, 104–106] although experience impacts its development [48, 59, 60, 107]. This lack of amygdala involvement does not prevent pups from learning about pain. Fear conditioning occurs in rat pups prior to PN10, including fetal pups when the amygdala basic circuitry is still maturing, although the pain must be intense (>1.0mA shock) and engage malaise learning or be mediated through a maternal fear pheromone [49, 68, 69, 108–112]. In contrast to the immaturity of the amygdala, in full-term human infants network analysis showed active connectivity between the PAG and ACC that served to dampen noxious stimulus elicited activity [113]. This is consistent with the expression of Fos at PN14 following an inflammatory injury [67], the analgesic effects of opioids injected into the PAG [114], and with manganese enhanced MRI (MEMRI) imaging that identified an “affective” pain circuit at this age that included the ACC, BLA, nAcc, PFC and PAG [85] at this age. Although we are unaware of fMRI studies of the hypothalamic PVN in children, this area has been explored extensively in rodents as a brain area important for the initiation of the HPA axis stress response. Although the HPA axis has complex controls, infant rats have a stress hyporesponsive period (SHRP), which, in part, is regulated by the hypothalamic PVN. The PVN is non-responsive to several stimuli, such as mild shock as shown here or to a potential predator [78, 115]. Originally thought to be completely inactive in infancy, critical work has shown that the PVN has a unique infant specific responsiveness that can be activated by ecologically relevant stressors such as cold and prolonged (>1hr) separation from the mother [116–119]. At PN10 there is sufficient stress hormone release to alter brain function and impact pups’ amygdala plasticity, fear and attachment suggesting an earlier end to the SHRP than was previously considered and an important role for maternal blockade of infant stress hormones defining neural networks [45, 46, 120].

### Infant effects of acute pain and social buffering on amygdala gene expression

In the microarray analyses, the presence of the mother induced fewer changes in amygdala gene regulation at PN8 than at PN12 with a lack of organized gene networks processes as evidenced by the lack of significant changes in Gene Ontology categories. This suggests that biological processes as a whole are not systematically altered in the PN8 pups as they are in the older pups. This lack of coherence at the youngest ages was reinforced by the IPA network analysis that found no significant canonical paths at PN8 except at very lenient criteria. When found at this lenient cutoff, paths were focused on inflammatory cells and tight junctions. These data suggest an immature circuitry at least in the amygdala, consistent with the Fos expression data here and the prior literature discussed above.

In sharp contrast to younger pups, PN12 pups that respond robustly to shock and returned to Control levels by mother’s presence, showed substantial changes in amygdala gene expression. The mother suppressed that activation, consistent with the prior literature [46, 76, 121]. Thus, the mother has a more significant effect on amygdala gene expression, both quantitatively and qualitatively, at PN12 compared to PN8, which is consistent with the ability of the mother to synchronize cortical activity at this age [102].

Taken together, these infant data highlight that neural circuits detect and respond to pain before functional inclusion of the amygdala, a structure well-established as an important component of the pain network in adults and older children. Overall, these infant data suggest that the young infant pain circuit continues to be unique, at least to some degree, compared to the older infant and adult networks and that pain experienced with the mother directly impacts the infant’s neural processing of both pain and the mother [50, 56, 59, 122–127].

### Adult outcomes are dependent on the age and the social context of infant pain treatment

To our knowledge, this is the first specific assessment of the enduring effects of pain experiences with and without the mother, engaging social buffering to reduce pain, on adult social behavior. These data on the social context of pain build on previous pain neurobehavioral programming data that has already clearly defined a sensitive period for pain programming until PN7-8 [71, 72]. Specifically, when an inflammation injury, such as carrageenan or complete Freund’s adjuvant, is used during the pain sensitive period, this injury can produce long terms effects of pain thresholds, typically either no change or hypoalgesia in baseline responsiveness and hyperalgesia on re-injury [reviewed in [128–131]]. Other models such as plantar incision, or repeated paw stabs have not been as well characterized but in general produce the same changes in pain sensitivity [24, 25, 132]. Although these models all include injury to the paw with subsequent testing in adults of the neonatally injured paw, the current studies applied shock to the tail. Of note, early PN5-9 repeated shock did not produce changes in thermal baseline thresholds, consistent with some studies [74, 133], but also opens the possibility that off-site testing of early injury may differ from that in which a paw is injured and later tested.

#### Adult thermal pain threshold following infant treatment

*PN10-14 Social buffering of pain*, *but not pain alone*, *induced hypoalgesia*. Infant shock without the dam did not change adult thermal thresholds: Adult shock only animals at neither infant age of exposure failed to alter response latencies compared to the Controls. However, treatment at PN10-14 with repeated Shock+mother pairings resulted in a mild but significant hypoalgesia for baseline withdrawal latencies. As we integrate our results into the pain experienced without social context literature, it is noteworthy that these data are consistent with those following infant foot stabs (punctate pain with defined intensity and time frame) with pain alone [24, 134]. Integration of our results with infant pain induced by inflammatory compounds is less conclusive, potentially due to myriad methods of delivery, age of treatment and control of pain duration, [some show hypoalgesia [71, 135, 136], some hyperalgesia [62, 135] whereas others have no change [73, 133]].

#### Adult thermal pain hyperalgesia induced by carrageenan following infant treatment

When the hindpaw was inflamed with carrageenan, the response quickened for all groups and the Shock only groups did not differ from controls, suggesting the carrageenan-induced hyperalgesia was not altered by the early painful experiences. This differs from other models using infant paw injury [71, 72, 137], possibly due to the duration of infant inflammatory pain or site of pain (e.g., paw vs tail). However, the presence of the mother during shock in the PN5-9 group resulted in a large reduction of the hyperalgesia. To our knowledge, this is the only example of an early pain-related non-drug manipulation that reduces adult pain on re-inflammation.

#### Carrageenan induced changes in Fos expression

Fos was assayed in multiple brain regions related to pain and pain affect in adults, focused on those that had been treated at PN5-9 since that was the group that showed altered hyperalgesia after carrageenan. Two patterns emerged: First, adults with early life Shock+mother pairing showed an overall decrease in Fos expression in multiple brain sites, including the prefrontal and cingulate cortex, nucleus accumbens core and basolateral amygdala. Second, shock with or without the mother reduced Fos expression in the ventral medial hypothalamus, lateral septum, and nucleus accumbens shell. The maternal presence as pups receive shock has been shown previously to attenuate the activity of myriad brain areas, including the hypothalamus, amygdala, ventral tegmental area and prefrontal cortex. This has been replicated in the amygdala of children [138]. That reductions persist beyond infancy and below control levels speak to the power of social buffering. Other regions such as the PVN and PAG showed no changes. Thus, early experience of shock with and without the mother had regionally specific changes in Fos activation.

#### Adult assessment of affective behaviors following social pain vs. pain alone

Although early life trauma impacts later life affect, assessing the role of social context has been challenging and not directly assessed. Within the human literature, repeated early life adversity associated with pain and trauma has ubiquitous impact on later-life brain function, including the amygdala, hippocampus and PFC [7–9, 18–22, 106, 139–141] although these studies likely include pain, neglect, malnutrition and stress within both social and nonsocial contexts. Animal studies suggest that with repeated exposures to trauma, neural alterations are causal for social deficits, although social vs. nonsocial contexts have not been explored [11–17, 23–25]. Here we built on these finding and directly compared social vs. nonsocial trauma and uncovered how the age of treatment and the social context of that treatment impacts later-life outcome.

Recently, a similar procedure has been used by our research team to assess the specific circuits impacted by repeated shock with the mother present versus alone (58). Over the course of repeated shock treatment with the mother present, we observed a gradual increase in atypical social behavior of pups towards the mom and a gradual decrease in the mother rat’s ability to buffer the neurobehavioral impacts of shock. These changes were linked with persistently elevated dopamine levels in the BLA that were causal in perturbing social behavior with the mother following shock treatment at PN14-15 and with peers at weaning age. None of these effects were observed in pups that were shocked alone. Using a similar procedure, the present studies expand on this, to highlight the specific role of maternal presence in buffering the neural pain response. In addition, the present studies demonstrate the impact of this repeated shock treatment on fear and pain lasting well into adulthood, beyond the social behavior network.

#### Adult social behavior

Pain and its treatment can alter social and emotional behaviors. Repeated heel sticks or intraplantar injection of carrageenan around the day of birth alter the later-life response to anxiety- and stress-provoking stimuli, although the specific response depends on the age and type of infant injury and whether the adult animals are stressed [24, 25, 61, 132, 142–144]. Moreover, ongoing bacterial infection, common in the NICU, combined with infant pain has long term consequences for pain affect that differ from either infection or infant pain itself [73]. Less is known about the long-term effects of non-pharmacological treatments used to dampen pain in the infant. Adult social behavior was decreased only in the Shock+mother condition, independent of age at time of treatment, but not in animals experiencing shock alone. This replicates the enduring impact of naturalistic pain with the mother [maltreatment from PN8-12 [91]]. Although shock alone did not impact social behavior, it is noteworthy that shock alone during that same age range resulted in an adult anxiety behavioral phenotype and gene expression patterns similar to those found here in the infant for Shock alone [90].

#### Adult fear behavior

There were also changes in fear behavior, but in contrast to social behavior, these were dependent on the age of infant treatment (PN10-14 only) and showed that infant shock exposure decreased fear responding (i.e., less hiding) regardless of social context (i.e., both Shock+mom and Shock alone). Thus, pain within a social and non-social context can have overlapping impact on some neurobehavioral systems.

#### Limitations

There are several limitations to this work. First, whereas we used shock which is easily controllable and can be precisely calibrated and is not prolonged, allowing more precise control of age of injury, shock is not ethologically relevant. However, its preciseness provides the advantage over inflammatory models such as carrageenan or complete Freund’s adjuvant, which can be more variable and long-lasting. In that sense, shock is more like heal sticks given experimentally or clinically. Moreover, we shocked the tail because we did not want to injure a paw in anticipation of the adult pain testing, whereas other studies injured one or more paws. The similarities and differences between tail injury and paw injury have not been systematically studied, although as referenced above, there can be global somatosensory effects of early localized injury [71, 145].

In addition, our sampling of brain regions for Fos analysis after acute injury in the infants was limited to three regions, which were based on adult and infant pain processing. Although we were able to identify unique regions for pain processing altered by maternal presence in the older infants, there remains a need for further study to either locate regions of activation at the younger age but not the older age, or to identify potential developmental continuities in pain processing. Likewise, our gene expression analysis was limited to the amygdala, largely because of its well-established role in maternal presence modulation of the infant’s response to threat and similar delayed development across infant humans and rodents. Thus, our findings of a relative lack of effects of the mother at PN8 was expected and we are providing additional neural network detail of that switch from PN8 to PN12.

#### Implications

Our data are consistent with the literature and add to our understanding of early life pain by suggesting that social buffering of early life pain has effects broader than simply blunting neurobehavioral response to pain—neural processing is blunted but unique neural signatures are recruited with distinct impact on the developmental neural trajectory. This has been suggested for sucrose, another nonpharmacological pain reduction technique used in the nursery [64–66].

Since social buffering of infant pain by the mother occurs across species, the rodent is an excellent model to explore this phenomenon in children. Since social buffering of pain is currently used as an intervention in the NICU to reduce the stress and pain of many of the minor, yet painful medical procedures it is important to understand its infant mechanisms and specifically question if it blocks the neurobehavioral negative impact of experiencing repeated infant pain [146, 147]. The present research begins to answer these questions by noting that mother’s presence did not simply block pain or block the neural response to pain. Indeed, whereas it is true that some markers of pain processing were reduced (i.e., reduced gene expression), it induced its own unique patterns of gene, neural and behavioral expression that transitioned across development. We are just beginning to understand the positive and negative outcomes of the mother’s buffering effects and the present research defines that maternal social buffering is not simply blocking pain, it is activating a unique neural signature in both infancy and adulthood supporting altered neurobehavioral development. Understanding this divergent neurodevelopmental pathway is critical as we define risks and benefits from medical interventions.

## Supporting information

S1 Fig Thermal withdrawal data from both paws. (PDF)

S2 Fig Fos expression in thalamus and PAG. (PDF)

S1 Table Behavioral data. (XLSX)

S2 Table Expression values from array. (XLSX)

S3 Table Ranked Products output. (XLSX)

S4 Table Ermine J inputs and outputs. (XLSX)
