# Supplementary figures and images for: Infant pain vs. pain with parental suppression: Immediate and enduring impact on brain, pain and affect
Source: PLoS One. 2023 Nov 16;18(11):e0290871. doi: 10.1371/journal.pone.0290871 (PMC10653509; doi:10.1371/journal.pone.0290871)

## PN 5-9

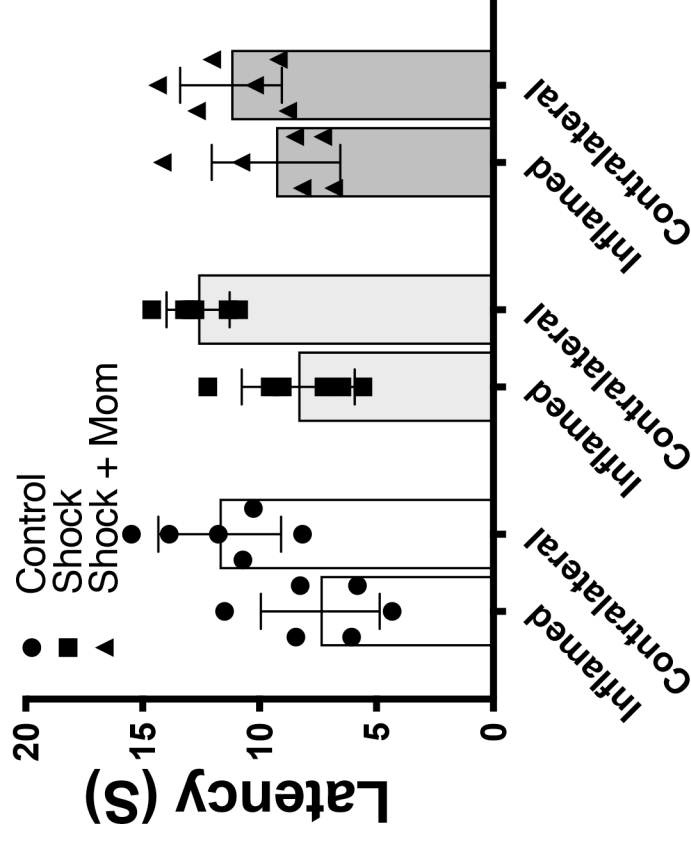

## Paw

Paw effect  $F(1, 15) = 92.3$ ,  $P < 0.0001$   
Paw X Exper. Tx Interaction  $F(2, 15) = 4.71$ ,  $p = .026$

## PN 10-14

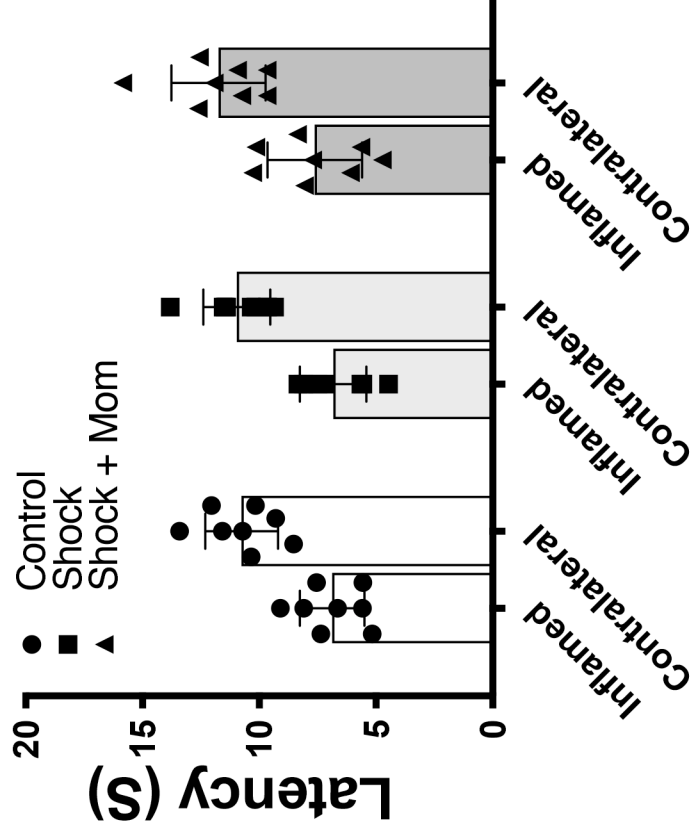

## Paw

Paw effect  $F(1, 21) = 124$ ,  $P < 0.0001$

Supplemental Figure 1

Supplement: S1 Fig — (PDF) [file pone.0290871.s001.pdf]

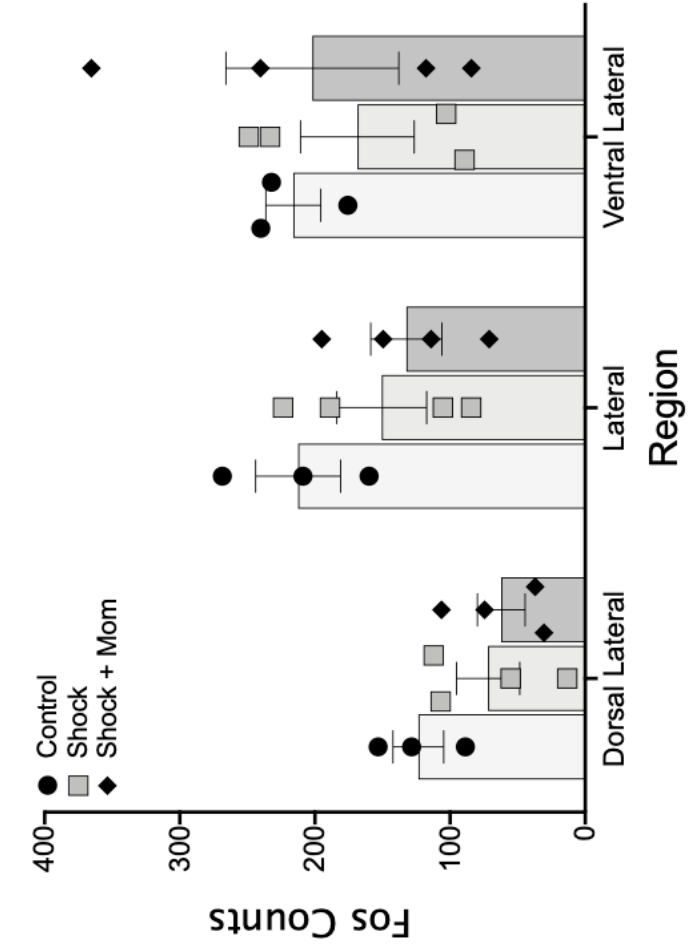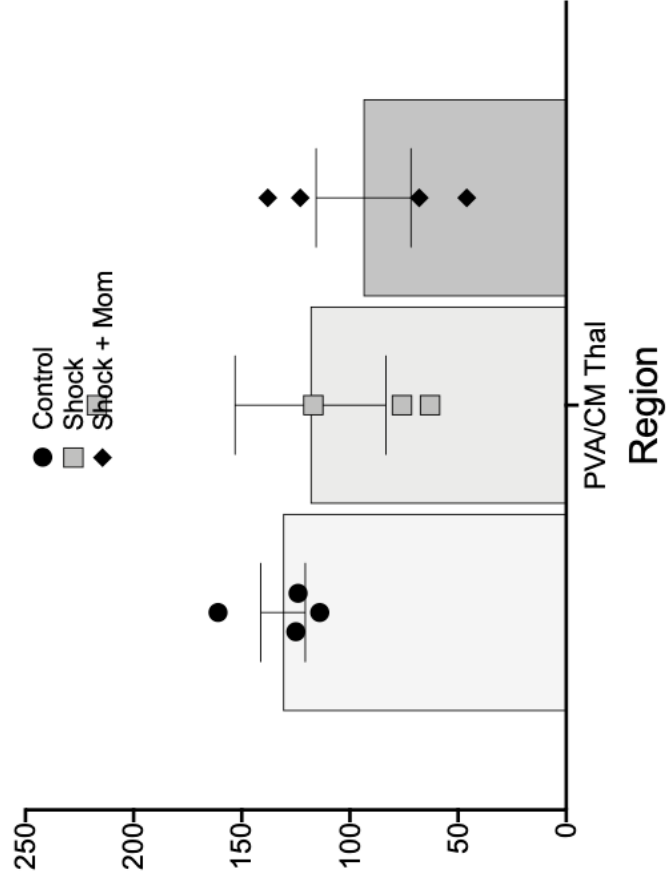

Supplement: S2 Fig — (PDF) [file pone.0290871.s002.pdf]
